# Supplementary material for: Are carbon emissions trading and green financial instruments synergistic? -Comprehensive quantitative research based on content analysis
Source: PLoS One. 2024 Mar 7;19(3):e0298601. doi: 10.1371/journal.pone.0298601 (PMC10919601; doi:10.1371/journal.pone.0298601)
Supplement: S1 Data — (ZIP) [file pone.0298601.s001.zip › Data/地级市Z_F工作报告.zip!/╝¬┴╓/═¿╗»╩╨/└┤╫╘╣1⁄2╓┌║┼í╛┬φ┐╦╩2╛▌═°í┐_2016.docx]

各位代表：

　　现在，我代表市政府，向大会作政府工作报告，请予审议，并请市政协各位委员提出意见。

　　一、“十二五”和2015年工作回顾

　　“十二五”是我市应对复杂挑战、经受严峻考验、取得重大成就的五年。五年来，我们在省委、省政府和市委的正确领导下，在市人大、市政协的监督支持下，坚持稳中求进工作总基调，统筹稳增长、调结构、促改革、惠民生，扎实进取，奋力拼搏，圆满完成了“十二五”规划确定的各项任务。

　　——综合实力显著增强。预计2015年实现地区生产总值1059亿元，是2010年的1.7倍，年均增长10.9%；地方级财政收入78.1亿元，是2010年的2.3倍，年均增长18.1%；规模以上工业总产值2118亿元，是2010年的2.3倍，年均增长18.1%；社会消费品零售总额479亿元，是2010年的2倍，年均增长14.2%；固定资产投资累计完成3853亿元，比“十一五”净增1914亿元，年均增长17.3%。医药产业产值年均增长29.4%，食品、建材、装备制造等支柱优势产业和战略性新兴产业、山区特色农业加快发展，新增规模以上工业企业144户、国家级高新技术企业13户、中国驰名商标11件，旅游收入是2010年的3.3倍，民营经济占GDP 的比重达到53%。

　　——改革开放成果丰硕。经济体制改革富有成效，社会体制、民主法制改革取得重大进展，生态文明、文化体制改革扎实推进，一些制约发展的深层次矛盾得到有效破解，开放型经济达到新水平。医药高新区晋升为国家级高新区，国家农业科技园区规划建设获得批准，7个省级开发区创新发展成果持续扩大。招商引资到位资金2860亿元，实际利用外资16.9亿美元，一批国内外知名企业落户通化。境内外上市企业达到19户。外贸进出口总额累计完成29.4亿美元。通道建设实现重大突破，至沈阳、丹东、长春3条高速公路全线贯通，通丹铁路建成通车，通化机场实现通航，通化的区位优势明显提升。

　　——城乡面貌发生较大变化。市区城建投资210亿元，完成50多项重点工程，城市功能和品位加速提升。“暖房子”工程改造面积774万平方米，获“中国人居环境范例奖”；拆迁棚户区50112户，改造棚户区580万平方米；集中供热率、供水普及率、垃圾处理率分别达到95%、92%、96.8%，燃气用户发展到10.2万户；改造建设了118条街路巷路、10座桥梁、6个公园广场，新增绿地面积447万平方米；江南新区初具规模。县域综合发展能力持续增强，特色鲜明的县城形象进一步展现。实施新农村建设项目2.3万个，建成省级示范村、重点村275个，修建农村公路1988公里、江河堤防256公里，植树造林60.5万亩，连续61年无重大森林火灾，农村人居环境明显改善。

　　——人民生活水平进一步提高。坚持实施民生工作计划，每年为群众办100多件实事。城镇居民人均可支配收入由16704元增加到28436元，年均增长11.2%；农村居民人均可支配收入由6572元增加到10621元，年均增长10.1%。企业退休人员养老金、城市和农村低保标准分别提高60.7%、73.1%和84.4%，城乡养老、城镇医疗、新农合保障实现全覆盖，工伤、生育保险和住房公积金参保人数分别增长50.4%、34.4%、19.2%，全方位救助体系基本形成。保障性安居工程、农村危房改造全面推进，24.8万户住房困难群众居住条件得到改善。解决了56万农村人口的饮水安全问题。教育、卫生、体育事业长足进步，文化事业和文化产业蓬勃发展，人民群众幸福感进一步增强。

　　各位代表！刚刚过去的2015年，面对经济下行压力加大的外部环境和艰巨繁重的改革发展稳定任务，我们强力推进振兴发展落实年攻坚战，抓招商、落项目、保运行，主动作为，精准发力，全市经济社会发展稳中有进、总体向好。预计地区生产总值增长7%，固定资产投资增长12%，社会消费品零售总额增长9%，规模以上工业总产值增长10.2%。受“营改增”、部分重点税种税率调整等因素影响，地方级财政收入下降6.7%。一年来，脚踏实地做了以下主要工作：

　　一是全力稳定了经济增长。把稳投资作为稳增长的关键，加大重点产业、基础设施、社会民生等投资，组织开展重大项目巡检活动，亿元以上在建项目达到360个、新上项目占47.8%。修正医药科技产业园等40个10亿元以上项目开工建设，东宝120万瓶人血清白蛋白、天实5000万瓶大容量注射剂、金马异地新建等项目建成投产。招商引资到位资金831亿元，实际利用外资4.3亿美元，上海万峰等100个亿元以上项目落地开工。通梅高速公路竣工通车，通靖高速公路路基工程完工，通集、辉白高速公路开始征地拆迁。城市地下综合管廊列入全省试点，开工15.4公里，建成廊体3.5公里。全力以赴稳工业，定向精准施策，工业增速在全省“领跑”。工业企业扩能提升和“巨人”企业培育计划全面推进，131个技改升级工程启动实施，5户企业完成并购重组，30种新产品实现规模化生产，重点培育的60户“巨人”企业产值增长20%，修正药业进入中国企业500强。积极应对钢铁行业“严冬”，支持服务通钢解决生产经营难题，环保控制区拆迁安置工程顺利完成，冶金产业园建设扎实推进。7个江东老工业区搬迁改造项目列入国家支持范围。对中小微企业落实税收优惠政策、减免税收1.5亿元，扩大“营改增”试点范围、直接减税3.1亿元，新增本外币贷款105亿元，这些措施实实在在为企业减了负、输了血、增加了活力。

　　二是加快了产业结构调整。制定了绿色转型发展规划和医药健康、食品等5个产业发展实施意见。大健康产业布局全面展开，争取到了全省56%的医药健康产业专项资金等支持政策，生物医药集聚区试点通过国家评审，累计85户医药企业通过新版GMP 认证，医药产业产值达到1100亿元。医药高新区引进亿元以上项目27个，高端产业集聚平台作用开始显现。食品、建材、装备制造、化工产业增速均超过10%。益盛汉参等人参产业项目快速推进，快大人参交易市场投入运营，人参产业产值增长30%。鸭绿江河谷葡萄种植达到2.1万亩，汇源酒庄等项目建成投产，葡萄酒产业产值增长20%。通化山葡萄酒和万通药业荣登中国品牌价值榜。评选推出“通化旅游十景”，龙溪健康养生谷等20个重点旅游项目开工，集安市、辉南县分别被评为国家级生态文明旅游示范区、全国休闲农业与乡村旅游示范县，高句丽文物景区晋升5A级通过评审，旅游收入增长25%。红星美凯龙家居购物中心建成开业。注册开业电商超过1万户，"9521"买药网等电商平台上线运营，吉林省人参电子交易平台试运营，国富人参交易中心获省政府批准，通化县成为全国电子商务进农村综合示范县。三次产业结构调整到9:53:38，现代服务业投资比重比上年提高2个百分点。

　　三是推动了农业转型和县域发展。粮食生产再获丰收，总产量达到45亿斤。新建粮油高产万亩示范区41个、高标准基本农田10.7万亩、科学储粮仓5000套，完成水利工程61项。建设标准化牧业小区201个，畜禽规模化养殖比重达到85%。建成农特产业示范基地15个，食（药）用菌、鲜食玉米、山核桃等特色产业得到了较快发展。新增棚膜蔬菜面积4000亩。实施千万元以上农业产业化项目20项，规模以上龙头企业达到270户。新创绿色有机无公害农产品标识20个。新增家庭农场167个、农民专业合作社538个，土地流转面积达到128.5万亩，完成196个试点村土地确权登记工作。新农村建设取得实效，45个省级重点村完成省补项目70项。县域经济实现平稳较快发展。梅河口市扩权强县试点成效突出，发展活力显著增强。集安市推动开放发展，被省委、省政府确定为扩权强县试点。辉南县推动朝（朝阳镇）辉（辉南镇）一体化建设，产业集群集聚步伐加快。柳河县强化项目攻坚，特色产业、生态农业快速发展。通化县推进创新突破，生物制药等战略性新兴产业增势强劲。在全省县域发展综合考评排序中，梅河口市位列第1，通化县、柳河县、集安市、辉南县分列第9、11、18和20位，我市被评为组织推动工作先进单位。

　　四是加强了城市建设和管理。市区改造各类棚户区100万平方米，开工率居全省第1位，货币化安置拆迁户的做法在全省推广。综合整治老旧住宅小区24万平方米；通化大街工程开工，升级改造街路巷路36条，滨江堤防加固改造、二道江公铁立交等4座桥梁改造建设和绿化美化亮化、玉皇山公园改造等工程如期完工，佟佳江公园建成开放；城市二次供水改造3年任务2年完成，解决了9.4万户居民用水和水质安全问题，在全国作了经验介绍；72公里灰口铸铁燃气管网改造全面完成，燃气管网老化的安全问题得到有效改善；完成天然气门站及管线建设；新增供热面积100万平方米，热网监控和服务管理实现智能化；改造地下排水管网30.2公里、供水管网21公里；建成水冲公厕25座；开展文明交通城市创建活动，施划停车泊位9000个；新建公交候车亭22 个， 新增新能源公交车30 辆，成为全国公交“一卡通”互联互通试点城市；综合整治市容环境，严格城区文明祭祀管理，城市管理效能进一步增强。物业管理覆盖率达到65%。新型城镇化规划编制和中心城市总体规划评估工作全面完成，“城中村” 改造扎实推进。东昌区推动产城融合发展，城镇化建设迈出新步伐。二道江区狠抓城区和工矿区改造，公共服务设施明显改观。我市列入国家智慧城市试点，成为全国首批、全省首个全光网城市。

　　五是狠抓了生态环境保护建设。生态保护红线划定工作开始启动，创建全国国土资源节约集约模范市通过验收， 完成大气污染治理、污染物减排和重点流域污染防治项目69 个，累计淘汰燃煤小锅炉118 台， 注销黄标车1060辆，农村环境连片整治、哈泥河水源保护取得新进展。成功申报“中国野生山核桃之乡”，林地清收走在全省前列。完成了佛山等7 个地质灾害及矿山地质环境治理工程，南山生态移民工程启动实施，垃圾焚烧发电工程开工。通化县成为全省首个国家级生态县，柳河、集安成为省级生态县。新增省级生态乡镇7 个、生态村15 个， 石湖保护区晋升为国家级自然保护区。我市被确定为全国地级市唯一生态系统生产总值（GEP） 核算试点，被评为全国首批绿色发展优秀城市。

　　六是统筹推进了改革创新工作。市级层面106 项改革任务全部完成。市政府部门权力清单对外公布， 行政职权、许可事项分别削减44%、45.7%，部门和项目进厅率达到100%，向东昌区、二道江区和医药高新区下放管理权限26 项。实施重点项目“一站式办理、并联式审批”流程再造，平均压缩审批时间80 天。完成不动产统一登记职责整合和机构组建。推行“ 五证合一、一照一码”，新登记内资企业增长13.8%。建立并推进了国家新政策辅导机制，辅导各类企业2800 户次。在全省率先实施收购现房安置棚改户政策，回购存量商品房50 万平方米。制定了利用PPP 模式引入社会资本加快市政设施建设试行办法，29 个PPP 项目纳入全省储备库。信通融资担保公司和通粮集团组建工作扎实推进。嘉浩盈华股权投资基金进入通化。正和药业等5 户企业在“新三板”和上海股交中心挂牌。列入国家和省科技专项80 项，新增省级科技企业34 户、省级企业技术中心4户， 东宝药业被认定为国家技术创新示范企业。成功举办了长白山医药健康产业发展论坛和科技成果对接会等活动，累计与“十四校两所”签订科技战略合作协议，吉林大学技术转移中心和全省首家医药行业知识产权维权中心在我市成立。

　　七是着力保障和改善了民生。承诺的“十大民生工程”、107 件民生实事按期完成。城镇新增就业4.1 万人，农村劳动力转移就业25.7 万人。城乡居民养老保险实施并轨，企业退休人员月人均养老金增加214 元， 城市低保月标准、农村低保年标准分别提高到450 元和2600元， 新农合政府补贴人均增至380 元。机关事业单位养老保险制度改革全面启动。市区安置棚改居民7318 户，建设公共租赁住房6 万平方米，1.6 万户低收入住房困难家庭享受到租赁补贴。改造农村危房5822 户。协调发展各项社会事业，新建改建幼儿园59 所，改造了30 所学校的基础设施， 取消了普通高中择校生，5 个县（市、区） 通过国家义务教育均衡发展评估认定。通化师院应用技术学院获批成立；县级公立医院改革全面推开，基本公共卫生计生服务逐步实现均等化， 传染病防治水平稳步提升。完成259 所村卫生室标准化建设。我市被确定为全省中医药综合改革示范区；建成24 个社区老年人日间照料中心；成功举办通化市田径运动会和市民、农民文化节，社区活动室、乡镇文化站实现全覆盖。佟江文化创意产业园投入运营， 丸都山城遗址本体保护工程全面启动。实施了无线数字电视惠民工程。市全民健身中心开工建设，科技文化活动中心对外开放。组织开展了纪念抗日战争胜利70 周年系列活动，全省纪念东北抗联精神大会在我市举行。民族团结进步和妇女儿童、老龄、残疾人事业全面发展。被评为全国未成年人思想道德建设先进城市。军民共建再创佳绩，通过全国双拥模范城“八连冠”检查验收。

　　八是加强了政风建设和公共服务管理。扎实开展“ 三严三实” 专题教育， 组织开展了“机关作风建设年”活动，一批群众反映强烈的问题得到解决。积极推进法治政府建设，自觉接受市人大及其常委会的法律监督、工作监督和市政协的民主监督。办结人大代表建议75件、政协提案133 件。“ 六五” 普法全面完成。制定出台“三重一大”事项集体决策、重大决策专家咨询论证等制度规定35 项。政务微博“ 通化发布”获全省城市新媒体综合影响力奖。加强廉政建设， 落实行政监察， 完善审计监督，查处了一批违法违纪案件。化解信访积案1208 件，信访总量、进京非正常访数量分别下降16.5%和65%。市长公开电话受理群众来电5.5 万件。排查整改安全隐患10379 项，被评为全国安全生产月活动先进单位。“食品药品生产质量管理提升年”活动取得阶段性成果，各县（ 市） 均被评为全省食品药品安全示范县（市）。妥善处置"7•01" 集安重大涉外交通事故，得到了省委、省政府充分肯定和国务院应急办通报表扬。持续开展严打整治专项行动，深化反恐防暴和边境地区管控，圆满完成一系列重大活动安保任务， 确保了全市政治稳定、社会稳定、治安稳定和边境稳定。

　　各位代表！过去一年和“十二五”的成绩来之不易，这里饱含着省委、省政府对通化发展的亲切关怀，凝聚着全市广大人民群众的辛勤汗水。这些成绩是市委正确领导的结果，是市人大、市政协监督支持的结果。在此，我代表市政府，向全市各族人民，向人大代表、政协委员，向各民主党派、工商联、无党派人士和各人民团体，向驻通国省属单位、驻通部队和武警官兵，向所有关心支持通化发展的同志们、朋友们，表示衷心的感谢！在总结成绩的同时，我们也清醒地认识到存在的问题：经济发展面临较大下行压力，增长动力转换较慢，冶金行业和部分企业生产经营困难较多，财政收支矛盾凸显；服务业发展相对滞后，缺少大项目、好项目支撑，科技创新能力不强， 推动产业转型升级需下更大力气；区域发展不平衡、不协调，医药高新区辐射带动作用远未形成，市本级实力不强、亟待提速发展；创新社会治理面临许多新情况、新问题，不稳定因素依然较多，安全生产形势不容乐观，一些群众的利益诉求还没有得到很好解决，维护社会和谐稳定的任务还很繁重；政风建设有待进一步加强，一些公职人员懒政怠政、不敢担当、不善作为、效率不高、执行力不强，违法违纪案件仍有发生，软环境仍亟待改善。对于这些问题，我们将在今后工作中加倍努力，采取有效措施，认真加以解决。

　　二、“十三五”发展目标和重点任务

　　“ 十三五”是我市绿色转型发展的攻坚期，是全面建成小康社会的决胜期，尽管宏观形势和外部环境错综复杂，但重大机遇和有利条件很多。国家实施“一带一路”和新一轮东北振兴战略，全省加快东部绿色转型发展、打造医药健康支柱产业、建设借港出海南部大通道和鸭绿江国际经济带，为我们立足大格局、推动大开放提供了重要契机。我市优良的生态环境、优越的资源禀赋、独特的区位优势、坚实的产业基础、深厚的文化底蕴， 为绿色转型、全面振兴提供了重要支撑。我们要抢抓机遇，奋发图强， 扎实做好打基础、破难题、固根本、利长远、惠民生的各项工作，努力在新的历史起点上开创通化全面振兴发展新局面。

　　“十三五”时期，我市经济社会发展的指导思想是：全面贯彻党的十八届五中全会、省委十届六次全会和市委六届五次全会精神，认真落实中央“四个全面”战略布局、省委“三个五”战略部署，按照市委关于妥善处理“五个关系”、扎实推进“六个一”发展布局、着力打造“七个通化”的总体要求，以绿色转型、全面振兴为主题，以如期全面建成小康社会为目标，以创新、协调、绿色、开放、共享发展理念为引领， 推动新型工业化、信息化、城镇化、农业现代化同步发展， 推动医药高新区、中心城区和县域统筹协调发展，加快把通化建设成为吉林省东部绿色转型发展示范区、东北东部中心城市和国际医药健康名城。

　　“十三五”的主要奋斗目标：地区生产总值年均增长7.5%左右， 固定资产投资年均增长12%以上，规模以上工业总产值年均增长9%左右， 社会消费品零售总额年均增长10%左右，地方级财政收入年均增长5%。到2020 年， 地区生产总值和城乡居民收入比2010 年翻一番，确保如期全面建成小康社会。

　　实现上述目标，必须主动适应新常态，着力保持经济中高速增长，着力推动产业迈向中高端水平，着力提高发展质量和效益，着力形成平衡发展结构，着力改善生态环境，着力实现合作共赢，着力增进人民福祉，着力打造绿色通化、开放通化、活力通化、法治通化、和谐通化、幸福通化、美丽通化。

　　（一） 推进实现动力转换，保持经济稳定增长。突出投资拉动，扩大有效投资，构建以重点产业、基础设施、科技创新、生态环保、改善民生、社会建设“六大板块”为主的项目支撑体系，实施一批补短板、上水平、增后劲的重大工程， 固定资产投资累计超过5000 亿元。全力推进工业企业扩能提升和“巨人”企业培育计划，扩大技术改造和战略性新兴产业投资比重，培育产值超10 亿元的工业企业50 户，其中超20 亿元的20 户、超50 亿元的10 户、超百亿元的5 户，建成一批国内国际知名度高、竞争力强的旗舰企业。实施创新驱动，建立以高校院所为依托的产业技术创新联盟，重点建设省级以上特色产业示范基地，加快建设国家重点实验室、企业技术中心和中试中心、院士和博士后工作站等服务平台，培育创客空间等新型孵化器， 科技进步贡献率由50% 提高到55%， 努力把我市建成东北东部区域创新极和重要的科研基地、实践基地、科技成果转化基地。支持民营企业完善现代企业制度，加强技术创新、管理创新、商业模式创新。民营经济占GDP 的比重要达到64%。

　　（二） 加大结构调整力度，构建现代产业体系。突出发展大健康产业，以医药、食品、旅游三大支柱产业为支撑，以山区现代特色农业为基础，拓展领域、整合提升、互促共进，形成具有持续竞争力和支撑力的通化特色产业体系。推行“ 健康+N” 产业模式， 促进医药工业、医药商业与流通、医药包装、医疗器械、中药材规范化生产、医疗与健康服务协调发展， 巩固和扩大“ 药” 的基础、补上补齐“医”的短板，拉长健康产业链，打造具有国际影响力的现代医药健康名城。加大长白山绿色食品资源、特色农副产品开发转化力度，提高系列化、规模化、品牌化加工深度，建成全国重要的绿色食品和功能保健品产业基地。整体谋划推进大长白山旅游圈和鸭绿江旅游带，形成“大旅游、大产业、大发展”的格局，建成吉林省生态文化旅游第二极和东北亚国际重要旅游目的地。加快发展冶金、建材、装备制造三大优势产业，培育壮大新材料、新能源、节能环保等战略性新兴产业。实施品牌和标准化战略，创建全国知名品牌示范区。实施现代服务业提升工程， 建设东北东部商贸物流中心。加快发展养老养生产业，建设国际养生休闲度假基地。抓好“ 互联网+” 应用实施， 建成吉林省东南部云计算中心、东北东部信息交通枢纽。第三产业增加值占GDP 比重要达到43%以上。

　　（三） 加快农业转型发展，率先基本实现特色农业现代化。高质量建设国家农业科技园区， 形成北部粮牧、南部特产、中部城郊等3个特色农业功能区。实施通化大米、鲜食玉米等优质粮食丰产工程，推进发展粮改饲和种养结合循环农业，加快发展优质畜牧业、园艺特产业，扶持壮大产业化龙头企业。建设全国人参科研、种植、物流基地和人参精深加工中心，大力开发药食同源新产品，做大做强人参产业。加快建设鸭绿江河谷葡萄产区，做优做响通化葡萄酒品牌。着力发展中药材、林蛙、食（药） 用菌、蓝莓等系列产业，因地制宜发展红松果林、山核桃、大榛子等经济林木和林下经济。实施棚膜蔬菜工程，建成一批蔬菜标准化种植基地。培养新型职业农民，抓好农产品品牌培育、市场开发， 强化农业科技服务，健全农产品质量安全全过程监管体系。推行一县一特色、一乡一品种等特色农业发展模式，全面加快特色农业产业化进程，推动农村一二三产业融合发展，争创全省山区特色农业转型发展排头兵。

　　（四） 立足大开放格局，加快国际化大通道建设。着力实施吉林省东南对外开放战略，深度对接辽宁沿海经济带、环渤海和京津冀经济圈， 主动融入中韩自贸区建设， 加强与韩、朝、俄、日、蒙等东北亚地区合作，对接国际化， 把我市建成吉林省向南开放的重要窗口。全面提高交通基础设施内外联通水平，实现高速公路“县县通”、中心城市“全辐射”，争取通化至沈阳、丹东、长春、佳木斯、集安5 条快速铁路工程全部启动，推动通化机场扩建和升级口岸机场，打通吉林省东南部借港出海大通道，打造东北东部区域性立体交通枢纽。全面推进鸭绿江国际经济带核心区建设，加快建设以“通化国际内陆港务区、医药高新区、集安边境经济合作区，中韩医药健康产业园、台湾生物科技产业园，东北亚医药健康产品展示中心、跨境电子商务中心及长白山国际医药健康产业发展论坛”为核心的“三区、两园、两中心、一论坛”国际化平台，真正使我市成为东北东部经济带开发开放的示范区。大力开展招商引资，扩大对外贸易，引进内资年均增长12%， 实际利用外资和外贸进出口总额年均分别增长7%和5%。

　　（五） 推动新型城镇化， 促进区域协调发展。科学推进城镇体系建设，以完善体系功能推动新型城镇化。着力发展壮大通化都市区，推进中心城区改造提升，加快新区开发、新城建设，实施地下综合管廊、生态公园、东南环路等一批提升品位、惠及民生的重点工程，完善市政设施和综合交通网络，完成地下管网改造升级，推进市区与通化县、集安市一体化发展，把通化建成风光秀美、生活舒适的宜居山水城市。大力发展城区经济，以现代服务业为主要抓手， 抓产业、抓税源项目、抓后劲培育，做大总量、做强实力。举全市之力推进医药高新区跨越发展，将其打造成为全市体制机制创新的示范区、高端医药产业的集聚区、科技创新和转型升级的核心区、经济发展的重要增长极、新一轮振兴发展的排头兵，成为全国医药健康产业发展的一面旗帜。建设智慧城市，构建高速、移动、安全、泛在的新一代信息基础设施。实施“气化通化”工程，实现城市天然气管网全覆盖。完成水利工程143 项，形成防洪、供水、灌溉等保障体系。围绕大健康产业发展，统筹市区、县城和特色城镇“三大板块” 协调联动， 形成融合配套、错位分工、优势互补的区域发展格局。全面实施东昌老工业区和二道江工矿区改造建设，增强县城和重点镇集聚服务功能。县城建设要完善功能、提升品质，城镇建设要突出特色、打造亮点。加快培育“ 养生镇”、“ 旅游镇”、“ 生态镇”、“滑雪镇”、“人参镇”等特色小镇，以城镇化推动县域经济争先晋位、加快发展。

　　（六） 全面深化改革，加快体制机制创新。完善“四张清单一张网”，深入推进简政放权、放管结合和优化服务， 抓好行政审批制度改革， 打造一流发展环境。按照省里统一部署，推动集安扩权强县改革，扩大县（市） 经济社会管理权限，建立权责一致、责任和财力相匹配的城区经济社会管理体制。推动金融产品创新，积极引进各类金融机构。争取IPO 或转板上市企业增加3 户、“新三板”等场外交易市场挂牌企业突破30 户。推进不动产统一登记制度改革。打好国企改革攻坚战，规范有序发展混合所有制经济，切实解决厂办大集体改革等历史遗留问题。抓好国有林区和国有林场改革，促进林区经济社会全面转型。深化农村综合改革，加快土地所有权、承包权、经营权分置并行，开展农村土地经营权抵押贷款等多种融资模式，实施城乡建设用地增减挂钩，稳步推进集体经营性建设用地入市，实施水利管理、水权制度改革，增强供销社社会化服务功能，确保社会体制等领域改革取得重大进展。

　　（七） 加强生态文明建设，增强发展可持续性。实施绿色低碳产业发展、重大节能减排与污染防治、循环经济、生态区域保护、生态廊道建设、生态文化与基础建设“六大工程”，巩固提升生态资源优势， 加大水、大气、森林、土壤等生态修复治理力度，增强国家级自然保护区、森林公园和湿地保护区生态功能，完成哈泥河等饮用水源的生态环境综合治理，推动辉南县龙湾群、通化县石湖两大生态保护开发协作区建设，打造沿江沿山沿路生态长廊，构建科学合理的生态文明制度体系。单位GDP 能耗年均下降2.1%，城市生活污水和垃圾无害化处理率分别达到95%、97.3%，实现国家卫生城市、园林城市、森林城市和生态文明建设示范市创建目标，全面提升长白山林区生态屏障重要门户的功能作用，坚决留给子孙后代一个天蓝、地绿、水净的美丽家园！

　　（八） 加大民生改善力度，实现发展成果共享。城乡居民收入增速超过全省平均水平，城镇居民人均可支配收入达到41300 元， 农村居民人均可支配收入达到19000 元， 各项民生指标争取排在全省前列。打赢扶贫脱贫攻坚战，确保2018 年基本实现脱贫目标。落实就业优先政策，进一步增强社会保障能力。加快保障性住房建设，完成城市棚户区、“城中村”和农村危房改造。推进医养结合， 建设以居家为基础、社区为依托、机构为补充的多层次养老服务体系。提高教育质量，促进教育公平。大力发展学前教育，均衡发展义务教育，普及高中阶段教育，加快发展现代职业教育。支持通化师院转型发展。打造区域医疗卫生中心，建立覆盖城乡的基本医疗卫生制度和现代医院管理制度。提升人口素质， 促进人口长期均衡发展。繁荣发展文化事业和文化产业，建设长白山医药健康文化中心。争创全国双拥模范城“ 九连冠”。增强城乡社区服务功能，强化安全生产监督管理和平安通化建设，构建全民共建共享的社会治理格局，争创全国依法治市先进城市。

　　三、2016 年主要工作

　　认真落实中央和全省经济工作、城市工作会议精神，坚持稳增长、调结构、惠民生、防风险， 围绕去产能、去库存、去杠杆、降成本、补短板五大任务，促进经济社会平稳健康发展，努力实现“十三五”良好开局。预期地区生产总值增长7.5%左右，地方级财政收入增长2%以上，固定资产投资增长12%左右，社会消费品零售总额增长10%左右，居民消费价格涨幅控制在3%以内， 城镇登记失业率控制在4.5%以内，城乡居民人均可支配收入与经济增长同步，节能减排完成省下达的指标任务。

　　（一） 全力推进项目建设和招商引资。新建续建亿元以上项目238 个、10 亿元以上项目45个，固定资产投资达到1100 亿元。开工建设东宝生物医药产业园、众方医药健康产业园和万通中药新药等新项目，加快实施修正科技产业园、紫鑫异地改造等续建项目，推动东宝6000万瓶甘精及门冬胰岛素注射剂、长龙3 万箱海昆肾喜等项目建成投产。立足大健康、大生态、大旅游、大枢纽等生产力布局，加强项目谋划储备，争取更多项目列入国家和省计划盘子。有针对性地开展园区招商、专业招商、以商招商， 抓好“ 台资企业吉林行” 和“ 世界500 强企业走进吉林” 等重大活动招商引资，引进亿元以上项目300 个，引进资金760 亿元。抢前抓早，组织好项目、招商等“冬季行动”，新建、续建项目5 月份开工率要分别达到80%、90%。

　　（二） 促进工业稳定增长和产业转型升级。继续实施工业企业扩能提升和“巨人”企业培育计划，推进100 个重点技改项目、10 户企业并购重组、120 种产品创新， 技改投资占工业投资比重达到60%以上。推动工业提质增效，抓好改善消费品供给、降本增效、制造业升级3 个专项行动。制定降低企业成本的具体措施， 实行收费清单管理模式， 为企业减轻负担。加强银企对接、产需衔接和生产要素供应保障，搞好监测分析，稳定工业运行。规模以上工业总产值增长9%。

　　加快产业结构优化升级。启动大健康产业5 年行动计划， 谋划布局千亿级产业扩容集群项目。抓好30 个重大医药健康产业项目、10 个中药大品种二次开发，推进建设国家生物医药集聚区， 创建全省医药健康产业发展示范区，把“通化•中国医药城”长白山医药健康产业发展论坛打造成为国家级永久性论坛品牌。加快发展绿色食品和保健品产业，实施康美、美保等30 个超亿元功能食品项目，提高食品产业精深加工能力和附加值。实施通葡、通天等葡萄酒系列产品改造， 促进葡萄酒产业快速发展。加快建设冶金产业园， 支持服务通钢保生产、保稳定，协调消化钢铁产能。加快江东老工业区搬迁改造，续建装备制造产业园，石油化工机械改造项目投产达效。抓好玄武岩、石墨、钾长石、火山岩等战略性新兴产业集聚区建设， 开发高端产品。实施矿产资源“ 探宝工程”计划，推动石油项目勘探开发。启动消费品质量提升工程，继续开展“食品药品生产质量管理提升年”活动，全面提升通化产品和品牌声誉。

　　打好服务业发展攻坚战。加快建设旅游支柱产业，实施太极湾国际旅游度假区、罗通山遗址国家考古公园等重点旅游项目，争取龙湾等景区晋升5A 级景区； 启动冰雪旅游发展计划，规划建设果松国际滑雪场和金川滑雪游乐场，改建金厂滑雪场；整合旅游资源，策划覆盖全域、全时节的特色旅游线路，创建国家全域旅游示范区；加强旅游基础设施、标识建设和标准化服务管理，发展智慧旅游，开发特色旅游产品；强化宣传推介，抱团整体促销，组织好龙湾野生杜鹃花卉旅游节、鸭绿江国际枫叶旅游节等节庆活动， 扩大通化旅游的吸纳力、影响力。加快实施龙溪健康养生谷、贵隆养生养老等项目，推动养生养老产业化。提速发展商贸物流业，推进市区果蔬、粮油等交易市场建设，加快江南商圈业态提升；筹建通化综合物流港和铁路物流中心、城市配送物流中心，建成运营辉南鸿丰等物流园区；依托人参交易市场， 争取国家“ 市场采购贸易” 试点。大力发展互联网经济，加快“通药网”医药电子平台建设，提升吉林省人参电子交易和国富人参交易平台功能，推动电子商务进农村、进企业、进社区。优化开发结构， 发展房屋租赁，总结收购现房安置棚改户经验，积极消化库存商品房，促进房地产业健康发展。

　　（三） 全面做好“三农”工作。立足山地资源，突出发展特色农业。加快构建粮经饲三元种植结构， 重大粮食增产技术推广面积达到200 万亩， 正常年景下粮食产量稳定在36 亿斤，优质大米产量提高到70%，鲜食玉米种植达到7.5 万亩、加工能力达到11 万吨。新建标准化牧业小区40 个，完成无疫区建设任务。推动建设环城农业产业带、生态农业产业园、特色农业示范区，抓好20 个千万元以上产业化项目。发展壮大中药材、食（药） 用菌、蓝莓、山核桃、大榛子等特色产业，每个县（市） 建设1 个以上示范基地，特产业基地面积达到110万亩。新建棚膜蔬菜4000 亩。实施快大人参饮片加工等10 个亿元以上人参产业项目，推进人参种植规范化、质量标准化、加工精深化、产业集约化，人参产业产值增长31%。推动长白山林蛙产业园竣工投产， 开发林蛙系列产品。整合优选农特产品资源，集中进行品牌培育和宣传，扩大通化大米、长白山人参、集安山珍等品牌的知名度和市场占有率。新创绿色有机无公害产品标识20 个。推动耕地向专业大户流转， 促进农业规模化经营。发展壮大家庭农场、农民专业合作社、休闲农庄等新型农业经营主体，完善农村市场营销网络。加强城镇规划设计，搞好金厂省级城镇化试点，启动示范镇、重点镇建设和扩权试点，打造一批生态特色小镇。深入实施“百村示范、全面提升”工程，建设45 个省级重点村。抓好农村环境连片整治，启动农村“厕所革命”，推动农药化肥施用量零增长，建成10 个省级美丽乡村，创建2万个美丽庭院、2 万户干净人家。打响精准扶贫、精准脱贫攻坚战，实施单位包村、干部包户、党员参与帮扶，完成农村人口脱贫1.18 万人。

　　（四） 加快基础设施和城市建设。通靖高速公路建成通车， 通集、辉白高速公路全线开工，做好集安至本溪高速公路前期工作，加快建设通化大街和西昌、五月花互通立交工程。推动通沈高铁和通化至佳木斯、四平快速铁路完成前期工作， 同步筹划通化火车站搬迁改造，争取尽早开工。谋划推进通丹、通集快速铁路工程，做好通化机场扩建和口岸机场争取工作。新建柳河风电工程。抓好江河堤防整治等45 项水利工程。筹划实施城区水源取水口迁移及第二净水厂、应急备用水源和天然气置换工程。

　　加大城市建设和管理力度。坚持规划先导、建管并重，更加注重城市品质，更加注重市民意愿，着力提升城市功能和宜居水平。市区改造棚户区67 万平方米，综合整治老旧住宅小区19 万平方米，加快二道江棚改小区配套设施建设。实施官道岭拓宽改造，启动红旗桥东侧立交工程，升级改造3 座危桥和30 条主次街路。加快供热供气供水设施改造，完成调峰热源环保扩能、12 座换热站新建、24.2 公里老旧燃气和供水管网改造工程。新建续建城市地下综合管廊， 争取国家管廊试点和海绵城市试点。实施污水处理厂二期扩建，治理35 公里排污沟壑， 新建3 座强排泵站、22 座水冲公厕。实施三期平湖改造， 改善提升城区水域景观带。高水准搞好绿化美化亮化工程。统筹实施吉林省东南部云数据基地及智慧城管、智慧交通等信息化应用工程。开展“城市建设管理提升年”活动。进一步加强物业管理。继续抓好文明交通创建活动， 改扩建光明路等停车场，增加一批停车位。推进“四城联创”，国家卫生城市、园林城市、全国生态文明建设示范市创建工作要取得实质性进展，国家森林城市要力争通过检查验收。

　　（五） 进一步深化改革开放。突出供给侧结构性改革，确保改革落点实、效果好。完善权力清单，建立责任清单、投资负面清单、财政专项资金管理清单和政务服务网。抓好行政审批制度改革，优化流程，提高效率。赋予医药高新区、东昌区、二道江区更加完整、更加规范的管理权限，实现事权、财权相匹配，促进城区加快发展。理清市县财权和事权关系，调动各方面积极性。深化国税、地税征管体制改革，完成金税三期工程建设。建立跨年度财政预算平衡机制，防范化解债务风险。健全国有资本监管运营体系，推进市属经营性国有资产统一纳入国资监管范围。实行统一的市场准入制度，实现企业注册全程电子化。实施PPP 项目13 个， 做大信通等投融资平台。推进农村土地确权、土地经营权抵押贷款试点和农业保险工作， 抓好27 个国有林场改革试点。深化教育、医疗、文化领域改革，深化公安机关管理体制和人民警察管理制度改革，稳步实施社会保障、事业单位、价格市场化、群众信访等改革。启动开展私营企业建立现代企业制度试点工作，争取纳入东北振兴国家民营经济改革试点。

　　加快新一轮对外开放。落实省委、省政府支持集安开放发展若干意见，抓好集安扩权强县247项政策承接工作，做好国家级边境经济合作区和保税区申报工作。推进建设“通白丹”经济协作区，推动东北东部经济带上升为国家战略，争取鸭绿江国际经济合作区先行先试，实现中朝界河大桥和公路口岸联运通行。规划筹建通化国际内陆港务区，逐步构建集保税加工、现代物流、产业合作、科技创新于一体的开放合作区。加快建设中韩医药健康产业园、台湾生物科技产业园，推动各类开发区提速发展。医药高新区地区生产总值增长10%，开工、投产亿元以上项目超过30个。加强医药、食品、钢铁等出口基地建设，把更多的通化地产品推向国际市场。

　　（六）推动创新发展。申报60项国家和省科技专项，筹建产业创新联盟和医药研发及中试中心，用好市科技成果交易中心和省科技大市场，加快院士工作站和科技企业孵化器建设，与中关村和“十四校两所”搞好产学研用合作对接，推进重大科技成果转化。创新金融服务，积极引进各类金融机构，大力发展产业金融，开展好“助保金池”、“缴税贷”、“税易贷”等业务，支持银行扩大信贷投放规模，新增贷款达到110亿元。鼓励保险资金投向重大项目和基础设施。抓好企业上市，力争5户企业在“新三板”等资本市场挂牌融资。继续实施国家新政策辅导机制，举办双导师制硕士研究生班，全面开展“互联网+小微企业”行动，推进大众创业、万众创新。大力培养和引进领军型企业人才、创新型科技人才和高素质专家型人才，加大应用型、复合型、技能型人才培养和引进力度。重视企业家队伍建设，尊重企业家、关心企业家，努力让所有企业家和创业者在通化大地上都有施展才华、发展事业的广阔天地。

　　（七）抓好生态环境保护建设。高度关注空气质量，开展建筑和道路施工扬尘、城市裸露渣土和交通扬尘及燃煤、机动车排放、餐饮油烟污染等5个专项治理行动，继续淘汰黄标车；加大对秸秆焚烧的治理和管控力度，抓好通化县秸秆青贮和生物质、菌基、制酒等综合利用试点，其他县（市）搞好两个以上综合利用试点。高度关注水质状况，加强哈泥河等水源地环境保护，加快建设城镇污水和垃圾处理设施；开展玉带河、通天河、金厂河等小流域治理，整治污水沟渠，改善城乡亲水环境。推行工业节能、绿色建筑、绿色交通计划，推广循环经济和清洁生产。完成垃圾焚烧发电主体工程和设备安装。鼓励发展林下经济、沟域经济，植树造林10万亩，清收林地2.6万亩，标准化绿化村屯76个。加快矿区生态和地质环境综合整治。划定生态保护红线，加强自然保护区、国家森林公园和湿地公园等重要生态区域保护，推进保护区重要区域生态移民。基本完成南山生态移民工程。建设浑江、鸭绿江水系生态廊道，筹建哈泥河与浑江交汇口及金厂河口湿地公园，建设蝲蛄河湿地公园。继续开展生态系统生产总值（GEP）核算试点。要通过大力推进生态文明建设，真正让全市人民在青山绿水中欣赏美丽、在蓝天白云下享受幸福。

　　（八）加强以改善民生为重点的社会建设。带着感情和责任，切实加大民生投入，着力解决民生问题，让全市人民的生活每年都有新变化、一年更比一年好。落实市委确定的“十大民生工程”，办好100件左右民生实事。

　　城镇新增就业3.9万人，农村劳动力转移就业24万人。推进社保扩面和保费征缴，提高企业退休职工养老金水平和城镇医保、新农合补助标准，推进城乡低保统筹发展，实现社会保障“一卡通”。健全以“保基本、救急难”为核心的大救助体系，保障困难群众基本生活。发展医养结合型养老机构，加快社区居家养老服务中心和农村养老服务大院建设。加强残疾人基本公共服务。抓好保障性安居工程，为1.5万户住房困难家庭发放住房租赁补贴。强化物业管理，市区基本物业覆盖率提高到70%。继续推进“城中村”改造。新建公交候车亭30个，新增城市新能源公交车30辆。改造农村危房5000户。新建改造农村公路200公里。

　　协调发展各项社会事业。改扩建公办和奖补民办幼儿园30所，改造义务教育薄弱校舍6万平方米，推进中小学示范性综合实践基地、市一中教学楼工程建设。筹建通化医药健康职业技术学院。抓好医疗资源整合，完善公共卫生服务体系，加快多元化办医进程。继续实施传染病防治工程。新建市医院门诊楼，建成市结核病防治所，完成市中医院搬迁改造。落实好两孩生育政策。实施文化惠民工程，开办“健康大讲堂”，培育有医药城特色的医药健康文化氛围。推进无线数字电视工程建设。做好万发遗址、自安山城等文化遗产保护工作。推进城市规划展览馆建设。长白山民俗博物馆、市图书馆和朝鲜族艺术馆建成开馆。规划建设文化创意产业园。建成市全民健身中心，实现社区、乡镇、行政村公共体育设施全覆盖。备战好第十三届全国冬运会。推动民族团结进步。保障妇女和未成年人权益。加强国防动员和后备力量建设，推进军民深度融合发展。

　　提高社会治理水平。实施“七五”普法规划，做好地方立法工作，推进全国法治城市建设。加快信用体系建设，提高社会信用水平。加强矛盾纠纷排查调处，推动信访事项及时就地化解。创新立体化治安防控体系，依法打击各类违法犯罪。维护边境安全稳定。落实安全生产目标责任，强化重点领域专项整治，防范重特大事故发生。完善应急管理协调机制。抓好防汛抗旱、森林防火、地震防灾减灾等工作。健全覆盖城乡最严格的食品药品安全监管体系，保障人民群众“舌尖上的安全”。

　　（九）强化政风建设。适应经济发展新常态和从严治党新要求，市政府要致力形成“负责任、敢担当，重实干、抓落实，有能力、会干事，严自律、保干净”的政风，始终不渝推进法治政府、服务政府、效能政府、清廉政府建设。强化依法行政，做到心中有规矩。认真贯彻《法治政府建设实施纲要（2015-2020年）》，加快建设职能科学、权责法定、执法严明、公开公正、廉洁高效、守法诚信的法治政府。增强法治思维，依法决策、依法办事、依法接受监督。完善国家工作人员尊法学法用法守法制度，办好“政府法制大讲堂”。落实重大决策专家咨询论证和政府法律顾问制度，规范公正文明执法；强化从严治政，做到心中有戒尺。深入推进政府系统党风廉政建设，强化行政监察，推动公共资金、国有资产、国有资源等重点领域审计监督全覆盖，坚决查处腐败案件。严格执行中央八项规定和省委、市委具体规定，持之以恒纠治“四风”。以“三严三实”为行为准则，守纪律、讲规矩，干干净净做事，清清白白做人；强化政府服务，做到心中有人民。加大考核督查问责力度，坚决纠正不作为、乱作为，坚决克服懒政、怠政，坚决惩处失职、渎职。抓好软环境建设，真心实意帮助基层和群众解决问题；强化工作落实，做到心中有担当。树立大局观念和责任意识，保持昂扬向上、积极进取的精神状态，集中精力干事创业谋发展，主动作为，善谋善为，创造性地开展工作。雷厉风行抓落实，精益求精抓落实，坚持不懈抓落实，推动各项工作在全省“争一保二不出三”。

　　各位代表！新的一年开启新的希望，新的征程承载新的梦想。让我们在市委的正确领导下，在市人大、市政协的监督支持下，动员组织全市人民，夙夜在公，真抓实干，为如期实现全面建成小康社会宏伟目标而努力奋斗！
